# On the Relationship between the Macroevolutionary Trajectories of Morphological Integration and Morphological Disparity

**DOI:** 10.1371/journal.pone.0063913

**Published:** 2013-05-17

**Authors:** Sylvain Gerber

**Affiliations:** Department of Biology and Biochemistry, University of Bath, Bath, England; Ludwig-Maximilians-Universität München, Germany

## Abstract

How does the organization of phenotypes relate to their propensity to vary? How do evolutionary changes in this organization affect large-scale phenotypic evolution? Over the last decade, studies of morphological integration and modularity have renewed our understanding of the organizational and variational properties of complex phenotypes. Much effort has been made to unravel the connections among the genetic, developmental, and functional contexts leading to differential integration among morphological traits and individuation of variational modules. Yet, their macroevolutionary consequences on the dynamics of morphological disparity–the large-scale variety of organismal designs–are still largely unknown. Here, I investigate the relationship between morphological integration and morphological disparity throughout the entire evolutionary history of crinoids (echinoderms). Quantitative analyses of interspecific patterns of variation and covariation among characters describing the stem, cup, arm, and tegmen of the crinoid body do not show any significant concordance between the temporal trajectories of disparity and overall integration. Nevertheless, the results reveal marked differences in the patterns of integration for Palaeozoic and post-Palaeozoic crinoids. Post-Palaeozoic crinoids have a higher degree of integration and occupy a different region of the space of integration patterns, corresponding to more heterogeneously structured matrices of correlation among traits. Particularly, increased covariation is observed between subsets of characters from the dorsal cup and from the arms. These analyses show that morphological disparity is not dependent on the overall degree of evolutionary integration but rather on the way integration is distributed among traits. Hence, temporal changes in disparity dynamics are likely constrained by reorganizations of the modularity of the crinoid morphology and not by changes in the variability of individual traits. The differences in integration patterns explain the more stereotyped morphologies of post-Palaeozoic crinoids and, from a broader macroevolutionary perspective, call for a greater attention to the distributional heterogeneities of constraints in morphospace.

## Introduction

Heterogeneous patterning of morphospaces (quantitative state space representations of taxa relative to an underlying set of possibilities for morphological variation) has been frequently documented in clade-wide temporal studies, and is now widely acknowledged as a prominent feature of phenotypic macroevolution [1]–[7]. These heterogeneities are expressed in the spread and spacing of taxa in morphospace, as revealed by statistical measures of morphological disparity [8], [9]. Morphospace and disparity patterns may variously be the expression of functional factors, developmental constraints, historical contingency and/or stochasticity influencing the waxing and waning of taxa over the evolutionary dynamics of clades [10].

Although morphological disparity analyses have been undertaken primarily as a means to globally characterize patterns of stability and change of realized morphospace during the long-term history of clades (the magnitude of disparity**)**, disparity arguably also has an underlying, non-trivial structure. This structure potentially reflects aspects of the hierarchical organization of phenotypes into quasi-independent units of evolutionary transformation, i.e., evolutionary modules [11]–[14]. This near-decomposability of morphological phenotypes, as can be observed or inferred when quantifying morphological changes within evolving lineages, underlines patterns of differential integration within and among suites of phenotypic traits influenced by pleiotropic effects, developmental pathways and functional factors [15], [16].

In a macroevolutionary context, how phenotypic integration and modularity may actually be related to morphological disparity is an important but still largely unexplored question [17], [18]. For instance, might changes in morphological disparity characteristically result from the interplay of parcellation and integration of phenotypic organization (decrease or loss of correlation within primarily integrated set of traits leading to increased modularity and vice-versa)? Or might they result instead from intrinsic changes in the variational potential of a relatively constant number of modules? When are integration and disparity likely to correlate? Analogously, might disparity be operationally used as a meaningful proxy for modularity?

Here, I address some of these questions by quantifying the temporal trajectories of clade-wide measures of morphological integration in the Class Crinoidea (Echinodermata) over the Phanerozoic. The evolutionary history of crinoids is marked by two distinct radiations, occurring firstly in the early Palaeozoic (mainly Ordovician, ∼500–435 Myr ago) and secondly in the Triassic-Early Jurassic, as part of the recovery from the end-Permian mass extinction (∼251 Myr ago). Both radiations are characterized by rapid morphological diversifications at relatively low taxonomic diversity. Nevertheless, Foote [19] showed that post-Palaeozoic crinoids were morphologically less disparate than their Palaeozoic counterparts and also occupied a different, non-overlapping region of the morphospace. This distinct and more limited array of morphological designs perhaps suggests a different set of ecological opportunities [20] or internal constraints on the evolvability of crinoids. This case study, spanning more than 400 millions years of morphological evolution, enables one to portray macroevolutionary patterns of morphological integration and to contrast them with disparity profiles.

Given the temporal scale, the taxonomic level and the degree of morphological resolution, the temporal changes in the overall degree of integration do not focus on patterns comparable to those that are generally described at low taxonomic levels and concerned with small-scale aspects of organismal organization and variation. Rather, the evolutionary dynamics of integration quantified here is more closely related to the dimensionality of the crinoid morphospace *itself*, reflecting the highest levels of the hierarchical embedding of evolutionary modules within the crinoid body plan.

## Materials and Methods

The morphological dataset used in the present study has been compiled and regularly augmented by Foote [19], [21]–[25]. Its quality and adequacy for documenting evolutionary patterns in crinoids have been evaluated through numerous sensitivity analyses testing for potential biases induced by character selection and weighting, missing data, taxon sampling protocols, unequal time interval duration, morphospace dimensionality and disparity measures [19], [21]. The use of a different character-coding scheme applied to early Palaeozoic crinoids has also been tested and it provided results consistent with previous accounts [26]. The dataset includes 1032 species representing one species per genus per time interval. Each species is described by 90 discrete morphological characters offering a comprehensive coverage of the stem, cup, arm, and tegmen parts of the crinoid body (14, 40, 28, and 8 characters, respectively). See Foote [19] for further details on character definition and coding (data available in Appendix S1).

I followed two complementary approaches in order to allow the use of different measures of morphological disparity and integration. The first approach treats discrete characters directly and is hereafter referred to as the discrete character space approach; the second approach consists in extracting a dissimilarity matrix from the discrete character space using the mean character difference as the measure of morphological dissimilarity between two species [27] and then carrying out a principal coordinate analysis (PCoA) of this dissimilarity matrix. The first ten principal coordinates provide a fair representation of among-species dissimilarities and define the principal coordinate space explored in subsequent analyses.

With these two approaches, morphological disparity is measured as the mean pairwise dissimilarity and as the sum of univariate variances respectively, which are both standard indices of disparity relatively insensitive to sample size [28]. For discrete characters, I measured integration as the relative mean mutual compatibility. Two characters are said to be compatible if their state combinations do not necessarily imply homoplasy (e.g., for binary characters, not all four possible character state combinations 00, 01, 10 and 11 are found, so they can be mapped onto a tree without requiring convergence or reversal) [29]. In phylogenetics, compatibility analysis can be used to avoid overweighted correlated suites when selecting characters. For each time interval, I constructed a matrix of mutual compatibility, where the mutual compatibility of two characters *i* and *j* is defined as the total number of characters compatible with both *i* and *j* [30]. I then calculated the mean mutual compatibility and divided it by the maximum possible number of mutual compatibilities (i.e., total number of characters minus two). Hence, this measure of integration ranges from zero to one, respectively corresponding to low and high levels of correlation among characters.

For the continuous variables obtained via PCoA, I used the relative standard deviation of the eigenvalues of the correlation matrix proposed by Pavlicev et al [31] as a measure of morphological integration. This index also ranges from zero to one. If morphological integration is important, only a few dimensions are necessary to summarize most of the observed variation and the standard deviation of eigenvalues will be high because of the marked differences among them. Conversely, if morphological traits are weakly integrated, the standard deviation will be low because all eigenvalues will be roughly similar. These integration indices are therefore unrelated to the magnitude of disparity but instead describe its structure, that is, the dimensionality of the distribution of taxa in the morphospace.

The temporal partitioning of the morphospace into successive time intervals often leads to the extraction of matrices with more variables than individuals from the total morphological dataset. This “small *n*, large *p*” problem makes the sample correlation matrix an unreliable estimator of the population correlation matrix. Indeed, when the number of individuals becomes too small compared to the number of variables, the sample correlation matrix loses its full-rank and positive definiteness, thereby biasing the distribution of its eigenvalues and, in the present context, the measures of morphological integration. In addition, it has been shown analytically that the lower bound of the range of the standard deviation of eigenvalues for finite sample correlation matrices varies as (1/*n*)^1/2^ [32]. If not accounted for, this sample size effect can thus mislead the interpretation of temporal changes in integration, because the range of the index will vary as a function of taxonomic diversity. In order to circumvent these problems, I derived estimates of correlation matrices from a shrinkage procedure using the *R* package *corpcor* [33]. This approach allows one to obtain accurate, well-conditioned, and positive definite estimates of correlation matrices even for small sample sizes [34]. Based on simulations of random matrices of uncorrelated variables, I found that it also maintains the lower bound of Pavlicev et al.’s index close to zero down to sample sizes of about 15. Therefore, I chose to discard six time intervals with sample sizes lower than 15 to avoid any spurious estimates of integration: Early Ordovician, Late Permian, Triassic (two intervals) and Cenozoic (two intervals). The remaining time intervals have an average *p*/*n* ratio of 2.73 with a maximum of 6 (second time interval of the Cretaceous).

Comparison between morphological disparity and integration cannot be made directly because of the potential effect of trends and serial correlation inherent to most time series. To circumvent these effects, I used the generalized differencing approach [35], which consists of first detrending the time series by regressing their values against numerical time and then correcting for serial correlation by taking first differences (differences between adjacent values) modulated by the serial correlation coefficient (lag-1 coefficient). Correlation analyses between integration and disparity are performed on their generalized differences.

Finally, to trace the evolution of patterns of integration in greater details, I also used the metric recently proposed by Mitteroecker and Bookstein [36], the square root of the summed squared log relative eigenvalues, which provides a measure of distance between two covariance (or correlation) matrices. I computed all pairwise distances between the shrinkage estimates of correlation matrices corresponding to each time interval and then performed a principal coordinate analysis of the distance matrix obtained. This method enables to visualize the temporal trajectory of patterns of integration in the space of correlation matrices.

Assessing patterns of correlation and compatibility among characters can be hindered by the fact that species are not independent entities but parts of a hierarchically structured phylogeny resulting from branching evolution [37]. Unfortunately, in the absence of detailed phylogenetic hypothesis, it is not possible to correct for the non-independence of species by applying phylogenetic comparative methods. Nevertheless, in order to evaluate the potential effect of phylogenetic autocorrelation on estimates of integration, I applied the permutation-compatibility test for hierarchic structure in discrete character matrix [38]. This test compares the observed number of compatible character pairs with the null distribution obtained by permuting the original character matrix. If the observed compatibility is within the range of permuted matrices, then there is no (or little) phylogenetic signal in the data, and the observed patterns of integration are more likely to reflect secondary signals of correlated character changes.

Even though characters provide ecological and functional information about crinoid morphology, some also enable taxonomic distinctions within and among higher taxa [21]. To further ensure the robustness of the conclusions, all the above analyses have been run for the total morphological dataset, but also on a subset of 27 characters that are not taxonomically relevant (i.e., not used for diagnosing subclasses and orders; characters 1–2, 4–15, 21, 30, 47–48, 55, 57, 60, 62, 64, 69–71, 77), and should therefore not bear a strong phylogenetic signal. All statistical analyses were programmed and carried out in R (functions available in Appendix S2).

## Results

**Figure 1:**
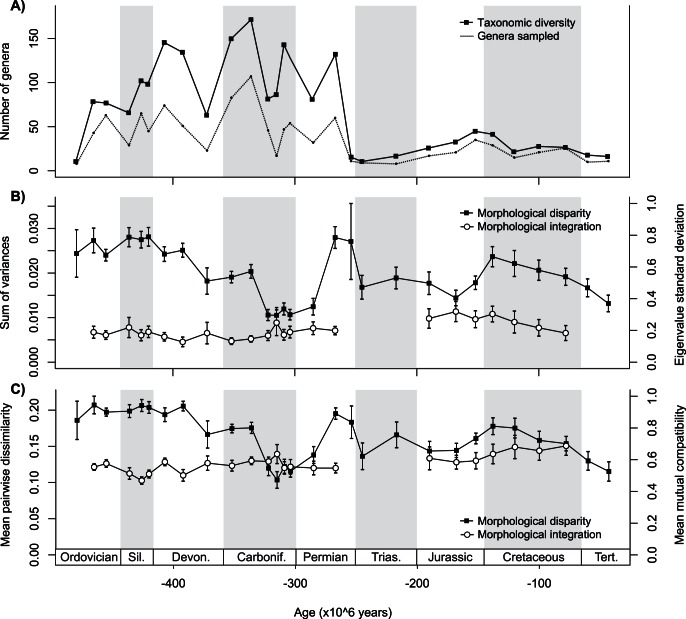
The temporal trajectories of taxonomic diversity, morphological disparity and morphological integration of Phanerozoic crinoids. (A) Number of genera known and number of species sampled per genus per stratigraphic interval. (B) Disparity measured as the sum of univariate variances; integration measured as the relative standard deviation of the eigenvalues of the correlation matrix. (C) Disparity measured as the mean character dissimilarity; integration measured as mean mutual compatibility. Error bars are bootstrapped standard errors. Because low sample sizes prevent from deriving reliable estimates of correlation matrices (see text), integration values are not presented for Early Ordovician, Late Permian, Triassic, and Cenozoic data. Whether based on the analysis of discrete or continuous variables, variations in the overall degree of integration do not appear to be associated with concomitant changes in disparity.

Figure 1 provides the curves of taxonomic diversity, morphological disparity and morphological integration for crinoids over the Phanerozoic, so as to examine the relative behaviours of these metrics, each emphasizing different aspects of biodiversity dynamics. Two complementary approaches are used in order to draw estimates of disparity and integration from both continuous and discrete character variables (Fig. 1A and 1B). As reported previously [19], morphological disparity shows marked variations over the period studied, most of them being decoupled from the rises and drops in taxonomic diversity. Contrastingly, indices of morphological integration measured as the relative standard deviation of eigenvalues and as the relative mean mutual compatibility appear to be fairly stable. Most increases and decreases in the overall degree of integration are not significant and do not appear associated with similar changes in the level of disparity. However, post-Palaeozoic crinoids on average display a higher degree of morphological integration than Palaeozoic crinoids for both measures of integration (*P*<0.01 in both cases with a Mann-Whitney *U* test).

**Figure 2:**
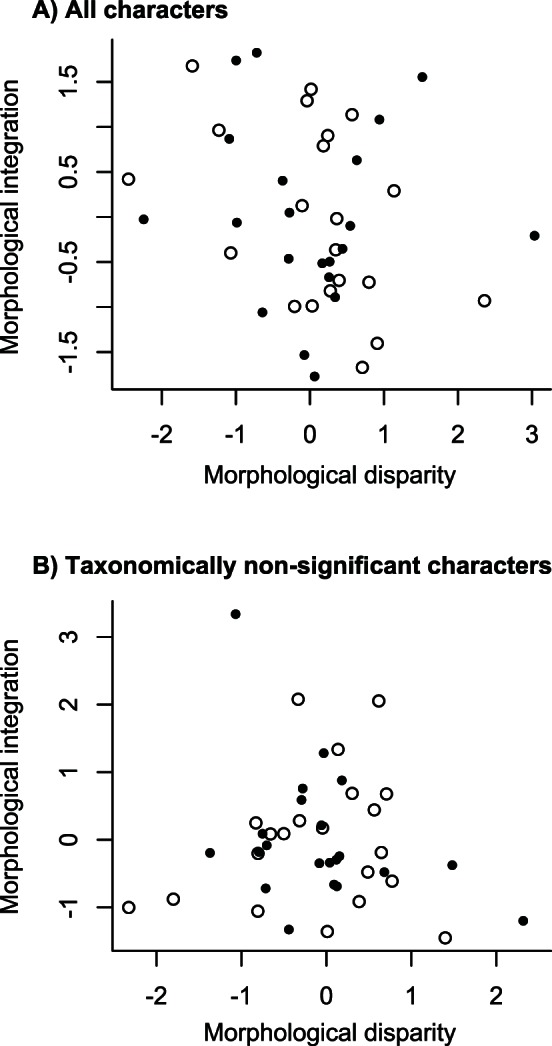
Correlation between temporal changes in disparity and integration. Spearmann’s rank correlation between level of morphological disparity and degree of morphological integration. (A) Generalized differences of morphological integration versus disparity for the PCoA-based approach (black circles; *r* = −0.118, *P* = 0.609) and the discrete character approach (open circles; *r* = −0.449, *P* = 0.042) when all characters are considered. (B) Generalized differences of morphological integration versus disparity for the PCoA-based approach (black circles; *r* = −0.340, *P* = 0.131) and the discrete character approach (open circles; *r* = 0.118, *P* = 0.609) when only taxonomically non-significant characters are considered (see text). In general, the amount of morphological disparity displayed by crinoids is not significantly correlated with the overall degree of integration among morphological traits.

I further investigate the relationships between degree of correlation among traits and level of morphological variety by calculating the correlation between the generalized differences of integration and disparity estimates (Fig. 2). Only the correlation between the mean mutual compatibility and the mean pairwise dissimilarity is significant (Spearmann’s *r* = −0.449, *P* = 0.042; Fig. 2A). Nevertheless, a permutation-compatibility test [38] detects a significant hierarchic structure in the dataset (as a whole and within individual time intervals), suggesting a phylogenetic signal potentially biasing estimates of integration (phylogenetic autocorrelation). I reran the same analysis on a subset of 27 characters of putatively low phylogenetic significance ([21] and see methods) and for which the permutation-compatibility test does not reveal significant underlying phylogenetic signal (Fig. 2B). Whether based on continuous or discrete character approaches, no significant correlation is observed between changes in disparity and integration (time series available in Appendix S3).

**Figure 3:**
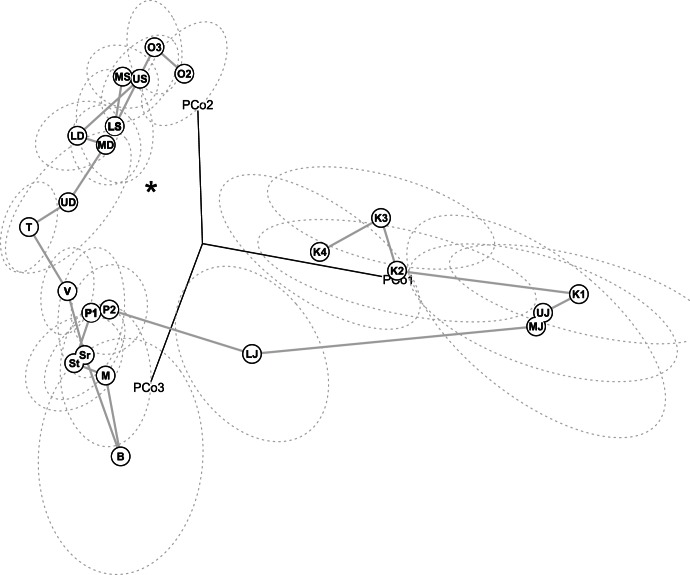
The temporal trajectory of integration patterns of Phanerozoic crinoids. The plot shows the first three principal coordinates of the space of correlation matrices. Each point corresponds to the correlation matrix of crinoids within a given geologic time interval (The correlation between pairwise Euclidean distances in the space of the first three principal coordinates and the actual distances between correlation matrices is 0.85). The grey line represents the temporal trajectory of correlation matrices from the Ordovician (O2) to the end of the Cretaceous (K4), and the asterisk gives the location of the identity matrix (i.e., a matrix with no integration among traits). Dotted lines are 68% confidence ellipses based on bootstrap resampling. Labels: O2 = Llanvirnian to lower Caradocian, O3 = remainder of Ordovician, LS = Lower Silurian, MS = Middle Silurian, US = Upper Silurian, LD = Lower Devonian, MD = Middle Devonian, UD = Upper Devonian, T = Tournaisian (Carboniferous, Mississippian), Sr = Serpukhovian (Carboniferous, Mississippian), B = Bashkirian (Carboniferous, Pennsylvanian), M = Moscovian (Carboniferous, Pennsylvanian), St = Stephanian (Carboniferous, Pennsylvanian), P1 = Asselian-Sakmarian (Permian), P2 = Artinskian-Kungurian (Permian), LJ = Lower Jurassic, MJ = Middle Jurassic, UJ = Upper Jurassic, K1 = Neocomian (Cretaceous), K2 = Barremian-Aptian (Cretaceous), K3 = Albian-Turonian (Cretaceous), K4 = Senonian (Cretaceous). The first principal coordinate separates Palaeozoic from post-Palaeozoic forms. The distribution of most Palaeozoic correlation matrices near the identity matrix emphasizes their homogeneous structure (roughly similar pairwise correlation among traits), whereas post-Palaeozoic correlation matrices display individuated blocks of correlated traits.

**Figure 4:**
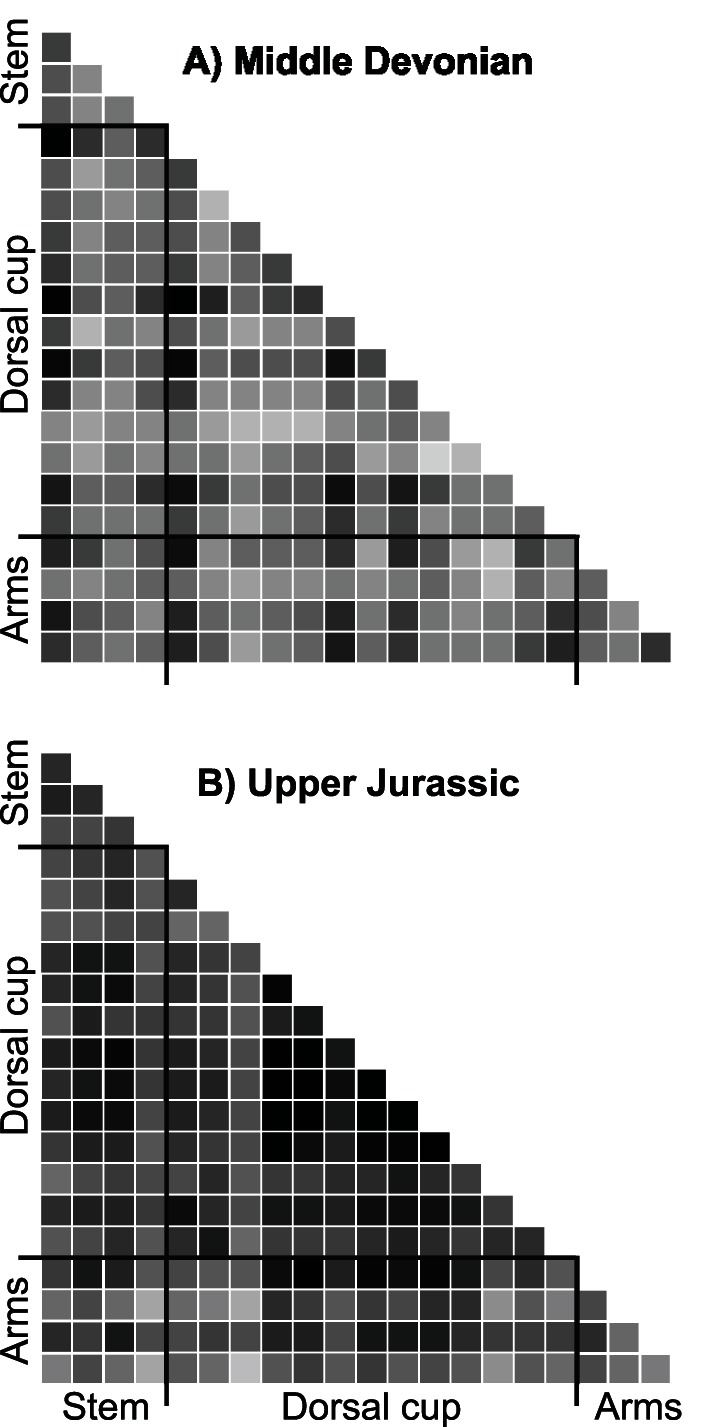
Matrices of mutual compatibility for Palaeozoic and post-Palaeozoic crinoids. These two matrices exemplify the differences in patterns of integration between (A) Palaeozoic (Middle Devonian, MD) and (B) post-Palaeozoic crinoids (Upper-Jurassic, UJ). The choice for these two time intervals has been driven by their location in the space of correlation matrix (separation along PCo1; see Figure 3) and the comparability of their sets of applicable characters (number and distribution over the whole character matrix). The gray-scale correlates with the strength of mutual compatibility (∼correlation): the darker the gray, the higher the compatibility. The comparison of these two matrices shows the overall stronger integration among characters within the post-Palaeozoic matrix and its heterogeneous structure with larger blocks of compatible characters (e.g., stem and dorsal cup characters, arm and dorsal cup characters).

Finally, I computed the pairwise distances among the trait correlation matrices associated with each geologic time interval so as to ordinate and visualize patterns of integration within the space of correlation matrices (Fig. 3). The temporal trajectory of correlation matrices follows a non-random pathway in this space, reflecting progressive but non-regular changes in patterns of integration across the Phanerozoic. The most striking feature of the distribution of these integration patterns is the clear separation of Palaeozoic and post-Palaeozoic patterns along the first principal coordinate of the space. The location of most Palaeozoic patterns in the vicinity of the identity matrix is indicative of homogeneously structured correlation matrices (i.e., all off-diagonal elements are of comparable magnitude), whereas post-Palaeozoic matrices tend to be more heterogeneously structured (unequal values of off-diagonal elements delineating blocks of variables; Fig. 4). The average distance among post-Palaeozoic patterns is significantly greater than that of Palaeozoic patterns (*P*<0.001; Mann-Whitney *U* test) despite the roughly equivalent duration separating successive intervals. This suggests greater magnitudes of transition between successive patterns of integration in post-Palaeozoic crinoids. Similar results are obtained when the space of correlation matrices is built from the set of taxonomically non-significant characters or from a drastic culling of data preserving only characters with less than five percent of missing data.

## Discussion

The present work examined large-scale patterns of evolutionary integration among morphological traits in crinoids and tested if and how changes in these patterns were associated with concomitant changes in the level of morphological disparity expressed by the clade. The analyses reveal relatively stable measures of the overall degree of integration despite marked temporal variations in taxonomic diversity and morphological disparity. Correlation analyses accounting for and limiting the effect of phylogenetic autocorrelation did not detect a significant one-to-one relationship between integration and disparity. Nevertheless, significant differences in the degree and pattern of integration are observed between Palaeozoic and post-Palaeozoic crinoids. Post-Palaeozoic crinoids have a higher overall degree of integration and occupy a different region of the space of correlation matrices. Their location indicates heterogeneously structured correlation matrices with larger blocks of correlated traits, which could explain the less disparate and more stereotyped post-Palaeozoic morphologies reported previously [19].

Hence, if the amount of morphological disparity does not appear to be conditional upon any given degree of overall integration, the results suggest that disparity is related to the modular nature of the correlation matrix, that is, to its pattern of organization into evolutionarily quasi-independent blocks of integrated traits. With regards to the two competing hypotheses presented in the introduction, the temporal trajectory of morphological disparity in crinoids would then be tied to changes in the pattern of correlation among traits rather than to changes in their individual variability.

In a study comparing the disparity levels of ecological and non-ecological (developmental) characters before and after mass extinctions, Ciampaglio [20] concluded that crinoid disparity patterns were mainly driven by the increasing structuring of ecological guilds rather than by developmental constraints. Nevertheless, his model of developmental constraints was focusing on upper limits for the level of disparity and not on biases in the spatial deployment of taxa in morphospace. Yet, developmental integration of traits and their dedication to specific functions generate evolutionary patterns of association and covariation among them, which shape the distribution of taxa in morphospace and the potential for evolutionary change [39]. The propensity of modular phenotypes to vary depends on the match between their developmental and functional modularity (i.e., the alignment of the genotype-phenotype map with the phenotype-fitness map; [40]). Specifically, if the pattern of developmental integration among traits coincides with their association to perform adaptive functions, evolvability is enhanced. Post-Palaeozoic crinoids derived from one family of Palaeozoic cladids [41] and their evolution has been characterized by an increased frequency of traits required for passive and active motility [42]. This has been interpreted as a response to increased interactions with benthic predators such as cidaroid sea-urchins [42], [43]. It is possible that these changes in predatory pressures may be responsible for the redeployment of traits into novel or modified functional complexes (increased aggregation of traits here). Then, the differences in evolutionary modularity documented between Palaeozoic and post-Palaeozoic crinoids potentially indicate a modification of the match between developmental and functional integration and the restricted range for trait covariation could explain the lower propensity to vary of post-Palaeozoic crinoids. Nevertheless, it is important to stress that current statistical indices of disparity are measures of observed macroevolutionary variation and therefore do not necessarily reflect the full potential to vary.

In summary, morphological disparity should be seen as more than a mere summary statistic of the amount of morphospace occupied. On the one hand, disparity reflects the building-up of the genealogical hierarchy over long timescales, with for instance the changing taxonomic composition of clades and the signature of mass and background extinctions. On the other hand, the behaviour of morphological disparity in face of these macroevolutionary phenomena is tied to the dimensionality of phenotypic variation constrained by the apportionment of variability among units of evolutionary transformation. Hence, the distribution and dynamics of taxa in morphospace should provide insights into the architecture of phenotypes and the constraints on their evolvability. This challenges the frequent conceptualization of morphospace as a homogeneous state-space. Such an interpretation of morphospace is unlikely to hold at the level of macroevolutionary phenotypic variation where development imposes a strong structure on the evolutionary accessibility of phenotypes (e.g. [44]–[46]). To different locations in morphospace are attached different sets of constraints and opportunities for phenotypic change in terms of probability, magnitude and directionality of evolutionary transitions. The morphospace is said to be structured [47], that is, patterns of phenotypic change are constrained by the location in the morphospace. This can be critical when comparing and interpreting the evolutionary dynamics of lineages originating in different regions of the morphospace. It does not mean that natural selection does not play any role at this scale, but rather that selection plays with a non-randomly distributed set of developmentally possible options in the vicinity of the evolving lineage [1], [48]. Further work is required to assess the relative role of selective pressures and developmental constraints in shaping patterns of diversification in the crinoid morphospace, for instance by conducting similar analyses at different temporal scales and taxonomic levels, in combination with an improved knowledge of crinoid development (e.g., [49], [50]). Even if developmental data are not directly obtainable for some groups and might imply hypotheses from comparisons with extant relatives, a greater attention to the organizational and variational properties of morphological phenotypes is necessary when constructing, exploring, and interpreting morphospaces. This is an important step to refine our understanding of the evolutionary history of higher taxa and of the processes driving macroevolutionary change.

## Supporting Information

Appendix S1 **Crinoid data (Foote 1999).** The file includes details of the stratigraphic intervals used, a description of the 90 discrete morphological characters, and the coding of these characters for the crinoid species retained in the analyses. See M. Foote, 1999. Paleobiology Memoir 1∶1–115 (supplement to Paleobiology vol. 25, number 2) for additional details (doi:10.1666/0094-8373(1999)25[1:MDITER]2.0.CO;2)
(TXT)

Appendix S2 ***R*** **functions.** The file includes the *R* functions for running disparity and integration analyses as described in the main text. Crinoid data are available in Appendix S1, *R* can be downloaded from http://www.r-project.org/. The shrinkage estimators of correlation matrices were obtained using the *R* package *corpcor* (http://strimmerlab.org/software/corpcor/). For additional details or questions: s.gerber@bath.ac.uk.
(DOCX)

Appendix S3 **Time series for morphological disparity and integration.** The file provides the numerical values of the temporal trajectories of disparity and integration throughout the Phanerozoic as displayed in Figure 1. It also includes the results when only the subset of taxonomically non-significant characters is used (see main text).
(XLS)

## References

[1] Alberch P (1980) Ontogenesis and morphological diversification. Am Zool 20: 653–667 doi: 10.1093/icb/20.4.653.

[2] Maynard-Smith J, Burian R, Kauffman S, Alberch P, Campbell J, et al (1985) Developmental constraints and evolution. Q Rev Biol 60: 265–287 doi:10.1086/414425.

[3] Raup DM (1987) Neutral models in paleobiology. In: Nitecki MH, Hoffman A, editors. Neutral Models in Biology. Oxford, UK: Oxford University Press. pp. 121–132.

[4] Arthur W (1997) The Origin of Animal Body Plans. Cambridge, UK: Cambridge University Press. 357 p.

[5] Foote MJ (1997) The evolutionary history of morphological diversity. Annu Rev Ecol Syst. 28: 129–152 doi: 10.1146/annurev.ecolsys.28.1.129.

[6] Gould SJ (2002) The Structure of Evolutionary Theory. Cambridge, MA: Harvard University Press. 1464 p.

[7] Erwin DH (2007) Disparity: Morphological pattern and developmental context. Palaeontology 50: 57–73 doi: 10.1111/j.1475–4983.2006.00614.x.

[8] Foote MJ (1993) Discordance and concordance between morphological and taxonomic diversity. Paleobiology 19: 185–204.

[9] Wills MA, Briggs DEG, Fortey RA (1994) Disparity as an evolutionary index: A comparison of Cambrian and recent arthropods. Paleobiology 20: 93–130.

[10] Jablonski D (2000) Micro- and macroevolution: scale and hierarchy in evolutionary biology and paleobiology. Paleobiology 26 (Suppl. to No. 4): 15–52 doi:10.1666/0094-8373(2000)26[15:MAMSAH]2.0.CO;2

[11] Olson EC, Miller RL (1958) Morphological Integration. Chicago, IL: University of Chicago Press (reprinted 1999). 376 p.

[12] Lewontin RC (1978) Adaptation. Sci Am 239: 156–169. 10.1038/scientificamerican0978-212 705323

[13] Wagner GP (1996) Homologues, natural kinds and the evolution of modularity. Am Zool 36: 36–43 doi: 10.1093/icb/36.1.36

[14] Raff RA (1996) The Shape of Life. Chicago, IL: University of Chicago Press. 544 p.

[15] Schlosser G, Wagner GP (2004) Modularity in Development and Evolution. Chicago, IL: University of Chicago Press. 600 p.

[16] Klingenberg CP (2008) Morphological integration and developmental modularity. Annu Rev Ecol Evol S. 39: 115–132 doi: 10.1146/annurev.ecolsys.37.091305.110054

[17] Chernoff B, Magwene PM (1999) Morphological Integration: Forty Years Later. In: Olson EC, Miller RL, editors. Morphological Integration. Chicago, IL: University of Chicago Press. pp. 319–353.

[18] Eble GJ (2004) The macroevolution of phenotypic integration. In: Pigliucci M, Preston K, editors. Phenotypic Integration: Studying the Ecology and Evolution of Complex Phenotypes. Oxford, UK: Oxford University Press. pp. 253–273.

[19] Foote MJ (1999) Morphological diversity in the evolutionary radiation of Paleozoic and post-Paleozoic crinoids. Paleobiology 25 (sp1): 1–116 doi: 10.1666/0094-8373(1999)25[1:MDITER]2.0.CO;2

[20] Ciampaglio CN (2002) Determining the role that ecological and developmental constraints play in controlling disparity: examples from the crinoid and blastozoan fossil record. Evol Dev 4: 170–188 10.1046/j.1525-142X.2002.02001.x. 12054291 10.1046/j.1525-142x.2002.02001.x

[21] Foote MJ (1994a) Morphological disparity in Ordovician-Devonian crinoids and the early saturation of morphological space. Paleobiology 20: 320–344.

[22] Foote MJ (1994b) Morphological disparity in Ordovician-Devonian crinoids. Contrib Mus Pal Univ of Michigan 29: 1–39.

[23] Foote MJ (1995a) Morphological diversification of Paleozoic crinoids. Paleobiology 21: 273–299 doi: 10.2307/2401167

[24] Foote MJ (1995b) Morphology of Carboniferous and Permian crinoids. Contrib Mus Pal Univ of Michigan 29: 135–184.

[25] Foote MJ (1996) Ecological controls on the evolutionary recovery of post-Paleozoic crinoids. Science 274: 1492–1495 doi: 10.1126/science.274.5292.1492 8929402 10.1126/science.274.5292.1492

[26] Deline B, Ausich WI (2011) Testing the plateau: a reexamination of disparity and morphologic constraints in early Paleozoic crinoids. Paleobiology 37: 214–236 doi: 10.1666/09063.1

[27] Sneath PHA, Sokal RR (1973) Numerical taxonomy. The principles and practice of numerical classification. San Francisco, CA: H. Freeman and Co. 588 p.

[28] Foote MJ (1992) Rarefaction analysis of morphological and taxonomic diversity. Paleobiology 18: 1–16 doi: 10.2307/2400977

[29] Camin JH, Sokal RR (1965) A method for deducing branching sequences in phylogeny. Evolution 19: 311–326 doi: 10.2307/2406441

[30] O’Keefe FR, Wagner PJ (2001) Inferring and testing hypotheses of cladistic character dependence by using character compatibility. Syst Biol 50: 657–675 doi: 10.1080/106351501753328794 12116937 10.1080/106351501753328794

[31] Pavlicev M, Cheverud JM, Wagner GP (2009) Measuring morphological integration using eigenvalue variance. Evol Biol 36: 157–170 doi: 10.1007/s11692–008–9042–7

[32] Wagner GP (1984) On the eigenvalue distribution of genetic and phenotypic dispersion matrices: Evidence for a nonrandom organization of quantitative character variation. J Math Biology 21: 77–95.

[33] Schäfer J, Strimmer K (2005) A shrinkage approach to large-scale covariance matrix estimation and implications for functional genomics. Stat Appl Genet Mol Biol 4: 32 doi:http://strimmerlab.org/software/corpcor/. Accessed 2013 Apr 25">10.2202/1544–6115.1175. Available: http://strimmerlab.org/software/corpcor/. Accessed 2013 Apr 25 10.2202/1544-6115.1175 16646851

[34] Ledoit O, Wolf M (2004) A well-conditioned estimator for large-dimensional covariance matrices. J Mult Anal 88: 365–411 doi: 10.1016/S0047-259X(03)00096–4

[35] McKinney ML, Owen CW (1989) Causation and nonrandomness in biological and geological time series: Temperature as a proximal control of extinction and diversity. Palaios 4: 3–15.

[36] Mitteroecker P, Bookstein F (2009) The ontogenetic trajectory of the phenotypic covariance matrix, with examples from craniofacial shape in rats and humans. Evolution 63: 727–737 doi: 10.1111/j.1558–5646.2008.00587.x 19087182 10.1111/j.1558-5646.2008.00587.x

[37] Felsenstein J (1985) Phylogenies and the comparative method. Am Nat 125: 1–15 doi: 10.1086/284325

[38] Alroy J (1994) Four permutation tests for the presence of phylogenetic structure. Syst Biol 43: 430–437 doi:10.1093/sysbio/43.3.430

[39] Gerber S, Hopkins MJ (2011) Mosaic heterochrony and evolutionary modularity: The trilobite genus *Zacanthopsis* as a case study. Evolution 65: 3241–3252 doi: 10.1111/j.1558–5646.2011.01363.x 22023589 10.1111/j.1558-5646.2011.01363.x

[40] Altenberg L (2005) Modularity in evolution: some low-level questions. In: Callebaut W, Rasskin-Gutman D, editors. Modularity: Understanding the Development and Evolution of Natural Complex System. Cambridge, MA: The MIT Press. pp. 100–128.

[41] Simms MJ, Sevastopulo GD (1993) The origin of articulate crinoids. Palaeontology 36: 91–109.

[42] Baumiller TK (2008) Crinoid ecological morphology. Annu Rev Earth Pl Sc. 36: 221–249 doi: 10.1146/annurev.earth.36.031207.124116

[43] Baumiller TK, Salamon MA, Gorzelak P, Mooi R, Messing CG, et al (2010) Post-Paleozoic crinoid radiation in response to benthic predation preceded the Mesozoic marine revolution. Proc Natl Acad Sci USA 107: 5893–5896 doi: 10.1073/pnas.0914199107. 20231453 10.1073/pnas.0914199107 PMC2851891

[44] Kaufman SA (1983) Developmental constraints: internal factors in evolution. In: Goodwin BC, Wylie CC, editors. Development and Evolution. Cambridge, UK: Cambridge University Press. pp. 195–225.

[45] Stadler BMR, Stadler PF, Wagner GP, Fontana W (2001) The topology of the possible: formal spaces underlying patterns of evolutionary change. J Theor Biol 213: 241–274 doi: 10.1006/jtbi.2001.2423 11894994 10.1006/jtbi.2001.2423

[46] Rasskin-Gutman D (2005) Modularity: jumping forms within morphospace. In: Callebaut W, Rasskin-Gutman D, editors. Modularity: Understanding the Development and Evolution of Natural Complex System. Cambridge, MA: The MIT Press. pp. 207–219.

[47] McShea DW (1998) Dynamics of diversification in state space. In: McKinney ML, Drake JA, editors. Biodiversity Dynamics. New York: Columbia University Press. pp. 91–108.

[48] Arthur W (2001) Developmental drive: an important determinant of the direction of phenotypic evolution. Evol Dev 3: 271–278. 11478524 10.1046/j.1525-142x.2001.003004271.x

[49] Hara Y, Yamaguchi M, Akasaka K, Nakano H, Nonaka M, et al (2006) Expression patterns of Hox genes in larvae of the sea lily *Metacrinus rotundus*. Dev Genes Evol. 216: 797–809. 10.1007/s00427-006-0108-1 17013610

[50] Shibata TF, Sato A, Oji T, Akasaka K (2008) Development and growth of the feather star *Oxycomanthus japonicus* to sexual maturity. Zoolog Sci. 25: 1075–1083 doi: 10.2108/zsj.25.1075 10.2108/zsj.25.1075 19267619

