# Supplementary material for: On the Relationship between the Macroevolutionary Trajectories of Morphological Integration and Morphological Disparity
Source: PLoS One. 2013 May 17;8(5):e63913. doi: 10.1371/journal.pone.0063913 (PMC3656834; doi:10.1371/journal.pone.0063913)
Supplement: Appendix S2 — R functions. The file includes the R functions for running disparity and integration analyses as described in the main text. Crinoid data are available in Appendix S1, R can be downloaded from http://www.r-project.org/. The shrinkage estimators of correlation matrices were obtained using the R package corpcor (http://strimmerlab.org/software/corpcor/). For additional details or questions: s.gerber@bath.ac.uk. (DOCX) [file pone.0063913.s002.docx]

**R functions used to perform analyses of morphological disparity and integration**

# Function calculating the dissimilarity matrix among taxa (see Foote 1999). X is the taxa*characters matrix, ‘ochar’ is the list of ordered characters.

MD <- function(X,ochar) {

w <- apply(X,2,max,na.rm=T) - apply(X,2,min,na.rm=T)

preochar <- rep(0,ncol(X))

preochar[ochar] <- 1

D <- diag(0,nrow(X))

ochar <- which(preochar==1)

no <- c(1:dim(X)[2])[-c(ochar)]

w[no] <- 1

pw <- combn(nrow(X),2)

for (i in 1:ncol(pw)) {

d <- (abs(X[pw[1,i],] - X[pw[2,i],]))

d[no][which(d[no]>1)] <- 1

d <- d*(1/w)

d <- sum(na.omit(d))/(length(na.omit(d)))

D[pw[1,i],pw[2,i]] <- d

}

D <- D+t(D)

list(D=D)

}

# For continuous variables, morphological disparity is measured as the sum of univariate variances. This is calculated as the trace of the covariance matrix.

# Function calculating the relative standard deviation of the eigenvalues of a given correlation matrix (based on Pavlicev et al. 2009, See also Wagner 1984). C is a p*p correlation matrix.

Pavlicev <- function(C) {

p <- ncol(C)

Ev <- eigen(C)$values

VarEv <- var(Ev)/p

SDEv <- sqrt(VarEv)

list(SDEv=SDEv)

}

# Function calculating the matrice of mutual compatibilities M, its corresponding dissimilarity matrix D and its Gower-transformed version T (see O’Keefe & Wagner 2001 for details)

COMPATIBILITY <- function(R) { # R is a discrete character matrix

n <- ncol(R)

C <- matrix(0,n,n)

cbn <- combn(n,2)

for (i in 1:ncol(cbn)) {

set <- cbind(R[,c(cbn[,i])])

cdt <- apply(set,1,sum)

val <- set[which(cdt==1),1]

ifelse(length(unique(cdt))==3 & length(unique(val))==2,C[cbn[1,i],cbn[2,i]] <- 0,C[cbn[1,i],cbn[2,i]] <- 1)

}

C <- C+t(C) ; M <- C%*%t(C) ; D <- matrix(1,n,n)-M*(1/(n-2)) ; diag(D) <- 0

gcm <- function(D) { # Gower-centered matrix

A <- -.5*D^2

ai <- matrix(apply(A,1,mean),n,n)

am <- matrix(mean(A),n,n)

d1 <- A-ai-t(ai)+am

}

T <- gcm(D)

list(T=T,D=D,M=M)

}

# Alroy’s approach to test for phylogenetic structure in a discrete character matrix X (Alroy 1994); nper=Number or permutations.

Alroy.test <- function(X,nper) {

obs <- comp.pairs(X)$C

n.dis <- c()

for (rep in 1:nper) {

perm.X <- X

for (i in 1:ncol(X)) perm.X[,i] <- sample(X[,i],length(X[,i]),replace=F)

n.dis <- c(n.dis,comp.pairs(perm.X)$C)

}

list(obs=obs,n.dis=n.dis)

}

# Required subfunctions:

compatible <- function(x,y) {

ic <- 0

comp <- apply(cbind(x,y),1,sum)

if (length(which(comp==0)!=0)) ic <- ic+1

if (length(which(comp==2)!=0)) ic <- ic+1

if (length(unique(x[which(comp==1)]))!=0 & length(unique(x[which(comp==1)]))!=1) ic <- ic+1

rep <- c()

ifelse(ic!=3 , rep <- "yes" , rep <- "no")

list(rep=rep)

}

comp.pairs <- function(X) {

cb <- combn(ncol(X),2)

C <- 0

for (i in 1:ncol(cb)) {

if (compatible(X[,cb[1,i]],X[,cb[2,i]])$rep=="yes") C <- C+1

}

list(C=C)

}

# A function that filters a character matrix to extract adequate submatrix for compatibility analyses

filter <- function(X) {

X[which(is.na(X))] <- 999

keep <- c()

for (cl in 1:ncol(X)) {

u <- X[,cl]

if (nrow(X)-length(which(u==999))>=4) {

if (length(unique(u[which(u!=999)]))==2) {

keep <- c(keep,cl)

}

}

}

if ( length(keep) != 1) {

x <- X[,keep]

x[which(x==999)] <- NA ; for (cl in 1:length(keep)) { x[which(x[,cl]==min(x[,cl],na.rm=T)),cl] <- 0 ; x[which(x[,cl]==max(x[,cl],na.rm=T)),cl] <- 1 }

}

if ( length(keep) == 1) { x <- matrix(0,nrow(X),ncol(X) )}

x

}

# Function calculating generalized differences of the time series y (x is time)

GD <- function(x,y) {

t <- lm(y~x)$coefficients

et <- y - (x*t[2] + t[1])

sc1 <- lm(et[2:length(et)]~et[1:(length(et)-1)])$coefficients[2]

dy <- as.numeric(c(et[1]*sqrt(1-sc1^2), et[2:length(et)]-sc1*et[1:(length(et)-1)]))

dy

}

# Function calculating the pairwise distances among a set of covariance matrices (Based on Mitteroecker & Bookstein 2009). X is an array of n covariance matrices

CD <- function(X) {

n <- dim(X)[3]

D <- matrix(0,n,n)

tgle <- combn(1:n,2)

for (i in 1:dim(tgle)[2]) {

A <- X[,,tgle[1,i]] ; B <- X[,,tgle[2,i]]

relEv <- eigen(solve(B)%*%A)$values # relative eigenvalues

D[tgle[1,i],tgle[2,i]] <- sqrt(sum((log(abs(relEv)))^2))

}

list(D=D+t(D))

}
